# Development of a Social Risk Score in the Electronic Health Record to Identify Social Needs Among Underserved Populations: Retrospective Study

**DOI:** 10.2196/54732

**Published:** 2024-03-12

**Authors:** Elham Hatef, Hsien-Yen Chang, Thomas M Richards, Christopher Kitchen, Janya Budaraju, Iman Foroughmand, Elyse C Lasser, Jonathan P Weiner

**Affiliations:** 1 Division of General Internal Medicine Department of Medicine Johns Hopkins School of Medicine Baltimore, MD United States; 2 Center for Population Health Information Technology Department of Health Policy and Management Johns Hopkins Bloomberg School of Public Health Baltimore, MD United States

**Keywords:** AI, algorithms, artificial intelligence, community health, deep learning, EHR, electronic health record, machine learning, ML, population demographics, population health, practical models, predictive analytics, predictive modeling, predictive modelling, predictive models, predictive system, public health, public surveillance, SDOH, social determinants of health, social needs, social risks

## Abstract

**Background:**

Patients with unmet social needs and social determinants of health (SDOH) challenges continue to face a disproportionate risk of increased prevalence of disease, health care use, higher health care costs, and worse outcomes. Some existing predictive models have used the available data on social needs and SDOH challenges to predict health-related social needs or the need for various social service referrals. Despite these one-off efforts, the work to date suggests that many technical and organizational challenges must be surmounted before SDOH-integrated solutions can be implemented on an ongoing, wide-scale basis within most US-based health care organizations.

**Objective:**

We aimed to retrieve available information in the electronic health record (EHR) relevant to the identification of persons with social needs and to develop a social risk score for use within clinical practice to better identify patients at risk of having future social needs.

**Methods:**

We conducted a retrospective study using EHR data (2016-2021) and data from the US Census American Community Survey. We developed a prospective model using current year-1 risk factors to predict future year-2 outcomes within four 2-year cohorts. Predictors of interest included demographics, previous health care use, comorbidity, previously identified social needs, and neighborhood characteristics as reflected by the area deprivation index. The outcome variable was a binary indicator reflecting the likelihood of the presence of a patient with social needs. We applied a generalized estimating equation approach, adjusting for patient-level risk factors, the possible effect of geographically clustered data, and the effect of multiple visits for each patient.

**Results:**

The study population of 1,852,228 patients included middle-aged (mean age range 53.76-55.95 years), White (range 324,279/510,770, 63.49% to 290,688/488,666, 64.79%), and female (range 314,741/510,770, 61.62% to 278,488/448,666, 62.07%) patients from neighborhoods with high socioeconomic status (mean area deprivation index percentile range 28.76-30.31). Between 8.28% (37,137/448,666) and 11.55% (52,037/450,426) of patients across the study cohorts had at least 1 social need documented in their EHR, with safety issues and economic challenges (ie, financial resource strain, employment, and food insecurity) being the most common documented social needs (87,152/1,852,228, 4.71% and 58,242/1,852,228, 3.14% of overall patients, respectively). The model had an area under the curve of 0.702 (95% CI 0.699-0.705) in predicting prospective social needs in the overall study population. Previous social needs (odds ratio 3.285, 95% CI 3.237-3.335) and emergency department visits (odds ratio 1.659, 95% CI 1.634-1.684) were the strongest predictors of future social needs.

**Conclusions:**

Our model provides an opportunity to make use of available EHR data to help identify patients with high social needs. Our proposed social risk score could help identify the subset of patients who would most benefit from further social needs screening and data collection to avoid potentially more burdensome primary data collection on all patients in a target population of interest.

## Introduction

Addressing social needs and social determinants of health (SDOH) challenges in the health care system has emerged as a key component of addressing health disparities [1]. Patients with unmet social needs continue to face a disproportionate risk of increased prevalence of disease, health care use, higher health care costs, and worse outcomes across a range of health-related domains [2-5]. Thus, health disparities cannot be resolved through traditional clinical interventions in the health care system. Targeted interventions to address social needs and SDOH challenges, especially among minority populations, are necessary to overcome widespread disparities [6].

The use of coding systems such as *International Classification of Diseases, Tenth Revision* (ICD-10) codes for social needs (ie, Z-codes) has increased in recent years, suggesting that clinicians and provider organizations are increasingly aware of social needs and SDOH challenges and the importance of screening for and documenting such needs [7]. Social needs are also extensively documented in unstructured electronic health records (EHRs), such as free-text provider notes [8-12]. Moreover, the rapid adoption of EHRs nationwide and the creation of associated health information technology tools have made it possible to use this growing body of data on social needs and SDOH challenges in risk prediction and adjustment models [13-21].

Some existing predictive models use EHR and administrative claims data on social needs to predict patterns of health care use, cost, and health outcomes [16,18-20,22]. Population-level data on community characteristics is a key component of understanding and addressing SDOH challenges and their impact on health care use, cost, and outcomes [23,24]. Therefore, some EHR-based models have developed linkages to these population-level data to better account for community-level information in their risk predictions [17,21,25-27]. Some existing models have also used the available data on social needs and SDOH challenges to predict health-related social needs [14,15] or the need for various social service referrals [13]. Despite these one-off research and pilot efforts, the work to date suggests that many technical and organizational challenges must be surmounted before SDOH-integrated health information technology solutions can be implemented on an ongoing, wide-scale basis within most US-based health care organizations.

Using both patient- and population-level data, we sought to develop a social predictive risk score based entirely on electronic information readily available within most health care delivery systems. Predictive models such as this could help providers to systematically identify patients at risk of having future social needs, who represent likely targets for further in-depth assessment of their social needs and ultimately potential referral to community-based organizations to address such needs. Using a systematic electronic case-finding screening approach such as this would help the health care system avoid burdensome and inefficient social needs assessment (eg, primary data collection from every patient at every visit).

## Methods

### Data Sources

This was a retrospective study using the Johns Hopkins Health System (JHHS) Corporation’s EPIC-based EHR structured data from July 2016 to June 2021. Based on the patient’s home address during the in-scope study periods, we linked community-level data (at the census block group level) from the US Census American Community Survey, 2018 five-year cohort [28]. We developed a prospective model (using current year-1 risk factors to predict future year-2 outcomes) within four such 2-year cohorts (ie, 2016-2017, 2017-2018, 2018-2019, and 2019-2020). Each cohort contained model predictors in the first year (2016 in the first, 2017 in the second, 2018 in the third, and 2019 in the fourth cohort) and model outcomes in the second year (2017 in the first, 2018 in the second, 2019 in the third, and 2020 in the fourth cohort). The overall data were randomly split into training and validation data sets (80% of the data were used for model development while the remaining 20% were used for validation). The final model was applied to the 2020-2021 cohort to evaluate its accuracy.

### Study Population

Adult patients aged 18 years or older at the time of entering the observation period who were alive at the end of the observation, had at least 1 eligible encounter in the first and second years of each study cohort, and had a valid address for linkage to population-level data were included in this study.

### Variable Selection

We identified variables with the highest potential impact on the health and social well-being of minority populations through a review of the literature and consultation with minority health, population health, and social needs and SDOH experts at JHHS. We also sought input from primary care providers and frontline workers, such as social workers and care managers, representatives of community-based organizations, and patients and their caregivers.

We identified a comprehensive list of predictors of interest available within the EHR’s structured data, including various patient- and community-level characteristics as well as health care use measures (Table 1) [29,30].

**Figure 1:**
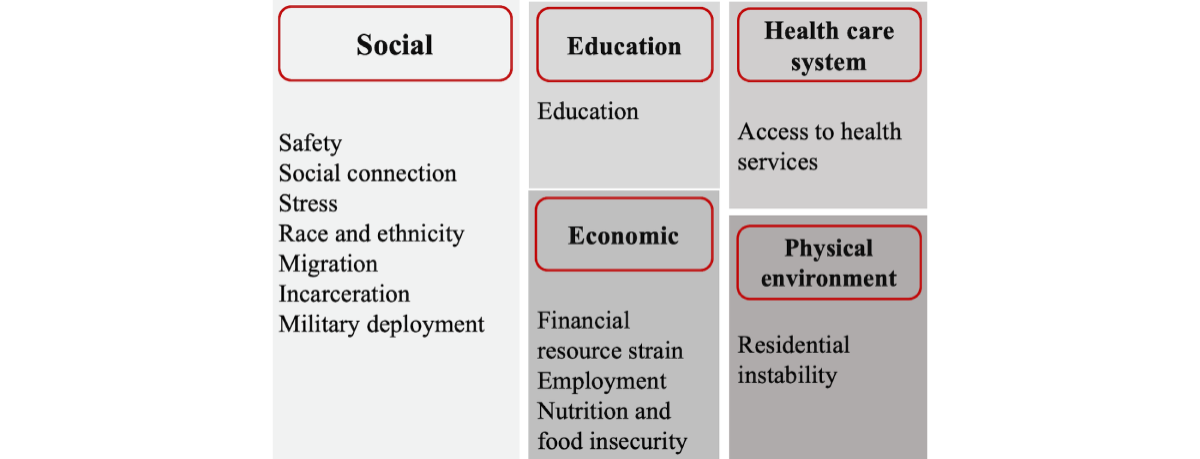
The framework for classification of social needs identified in the electronic health record (EHR) structured data.

To develop the variable on previous social needs, we obtained any ICD-10 codes presenting social needs using the “Compendium of Medical Terminology Codes for Social Risk Factors” developed by the Social Interventions Research and Evaluation Network [31] or any information on social needs available in the JHHS-EHR Wellness Registry, a data mart table in EPIC storing information related to general patient health, consolidated from many subject areas including social history and risk scores. After reviewing the classification of the ICD-10 codes by Social Interventions Research and Evaluation Network, we developed 13 subdomains and 5 domains of social needs (Figure 1).

We reviewed the ICD-10 codes and mapped each to a unique social need subdomain. We also reviewed available information on social needs in the EPIC Wellness Registry and selected variables corresponding to one of the 13 subdomains of social needs. We collapsed the responses available for each variable to generate a binary variable (“yes” or “no” indicator), suggesting the presence or absence of a social need. We defined previous social needs as a binary variable (“yes” or “no” indicator), suggesting the presence or absence of any corresponding mapped ICD-10 codes or any corresponding social needs identified in the EPIC Wellness Registry to 1 or more of the 13 social needs subdomains. We defined the outcome as a binary indicator of having a social need in the second year of each cohort (using the same logic as for the development of the predictor of social needs).

**Table 1:** Predictors of interest available within the electronic health record (EHR) structured data for inclusion in the generalized estimating equation model predicting prospective social needs for patients at Johns Hopkins Health System between 2016 and 2021.

Variable			Description
**Demographics**			
	Age (years)	Using the date of birth calculated at the time of entering the study cohort	
	Gender	Self-identified and reported at the time of entering the study cohorts	
	Race	Self-identified and reported at the time of entering the study cohorts	
	Preferred language	N/A^a^	
	Need for an interpreter	N/A	
**Previous health care use**			
	In-patient admissions	N/A	
	Emergency department visits	N/A	
**Previous social needs**			
	ICD-10^b^ codes	Documented using relevant ICD-10 codes	
	EPIC Wellness Registry	Documented in other structured social needs assessment fields, presented in the Wellness Registry Table	
**Clinical characteristics (derived from the Johns Hopkins ACG^c^ System version 12.0 [29], a widely used population-based predictive modeling and case-finding methodology)**			
	Number of chronic conditions	N/A	
	Medication active ingredients	N/A	
	Resource Utilization Band	Represents expected future use based on current morbidities	
**Neighborhood characteristics (associated with the person’s residence of longest duration)**			
	Area deprivation index	A composite measure allowing for the ranking of neighborhoods across the country by their socioeconomic disadvantage, reported at the census block group level [30]	

^a^N/A: not applicable.

^b^ICD-10: *International Classification of Diseases, Tenth Revision*.

^c^ACG: Adjusted Clinical Group.

The Adjusted Clinical Group (ACG) System Resource Utilization Band represent a simplified population segmentation system based on the overall morbidity burden of each patient. Representing expected future use based on current morbidities, the measure is calculated using all available ICD-10 codes for a person in the EHR during year 1, ranked from low to high according to the expectations of resources used during year 2.

### Statistical Analysis

We used a generalized estimating equation (GEE) model to predict prospective social needs, adjusting for the effect of the geographically clustered data as well as the effect of multiple visits for each patient (the records were clustered at the patient and 5-digit zip code level). The model selection was based on the goodness of fit test for GEE modeling, and the final risk score was composed using the variables identified as having the highest impact in the GEE model. We also validated the model using multiple denominators to ensure generalizability and retrained and tested the model for each subpopulation of interest (eg, individuals aged 65 years or older, racial and ethnic minority populations, and those living in the most and least disadvantaged neighborhoods).

### Ethical Considerations

The institutional review board of the Johns Hopkins Bloomberg School of Public Health reviewed and approved this study as exempt. The board approved the EHR data extraction for the secondary analysis of deidentified data.

## Results

### Demographics

The final study population included 1,852,228 patients in total. To be included in the sample, the patients had to be in at least 1 of four 2-year study cohorts (Table 2). The characteristics of patients across the study cohorts were comparable. Study cohorts included mostly middle-aged (mean age range 53.76-55.95 years across study cohorts), White (range 324,279/510,770, 63.49% to 290,688/488,666, 64.79%), and female (range 314,741/510,770, 61.62% to 278,488/448,666, 62.07%) patients from neighborhoods with high socioeconomic status (mean area deprivation index [ADI] percentile range 28.76-30.31).

Between 8.28% (37,137/448,666) and 11.55% (52,037/450,426)of patients across the study cohorts had at least 1 social need documented in the ICD-10 codes or EPIC Wellness Registry, with safety issues and economic challenges (ie, financial resource strain, employment, and food insecurity) being the most common documented social needs (87,152/1,852,228, 4.71% and 58,242/1,852,228, 3.14% of overall patients, respectively; Table S1 in Multimedia Appendix 1 provides details on the social needs domains across study cohorts). Between 18.67% (95,350/510,770) and 21.18% (95,393/450,426) of patients across the study cohorts had high or very high Resource Utilization Band, indicative of having a high disease burden, as reflected by many serious comorbidities.

**Table 2:** Characteristics of the study population for the development of the social risk score using electronic health record data at Johns Hopkins Health System between 2016 and 2021: overall and by 2-year enrollment cohorts (all characteristics are reported based on the first year of each cohort unless otherwise indicated).

Variables		Study cohort					Overall (N=1,852,228)
		2016-2017 (n=448,666)	2017-2018 (n=442,366)	2018-2019 (n=450,426)	2019-2020 (n=510,770)		
Age (years), mean (SD)		53.76 (17.65)	54.63 (17.43)	55.18 (17.25)	55.95 (17.34)	54.92 (17.43)	
Gender (female), n (%)		278,488 (62.07)	274,330 (62.01)	278,574 (61.85)	314,741 (61.62)	1,146,133 (61.88)	
**Race, n (%)**							
	White	290,688 (64.79)	285,558 (64.55)	289,611 (64.30)	324,279 (63.49)	1,190,136 (64.25)	
	Black	105,746 (23.57)	103,879 (23.48)	105,156 (23.35)	118,989 (23.3)	433,770 (23.42)	
	Other	52,232 (11.64)	52,929 (11.97)	55,659 (12.35)	67,502 (13.21)	228,322 (12.33)	
Preferred language (English), n (%)		430,331 (95.91)	423,396 (95.71)	430,024 (95.47)	465,173 (91.07)	1,748,924 (94.42)	
Interpreter needed (yes), n (%)		9,356 (2.09)	9,715 (2.20)	10,632 (2.36)	12,928 (2.53)	42,631 (2.30)	
Area deprivation index national rank, mean (SD)^a^		30.31 (23.6)	29.93 (23.46)	29.65 (23.24)	28.76 (22.81)	29.63 (23.27)	
**Health care use, n (%)**							
	Any in-patient admission	39,364 (8.77)	37,154 (8.4)	38,407 (8.53)	41,149 (8.06)	156,074 (8.43)	
	Any emergency department visits	71,737 (15.99)	69,436 (15.70)	69,649 (15.46)	77,308 (15.14)	288,130 (15.56)	
**Previous social needs, n (%)**							
	Year 1: ICD-10^b^ codes	37,137 (8.28)	50,332 (11.38)	52,037 (11.55)	57,170 (10.21)	191,693 (10.35)	
	Year 1: EPIC Wellness Registry	0 (0)	0 (0)	2 (0)	6,663 (1.3)	6,665 (0.36)	
	Year 2: ICD-10 codes	45,272 (10.09)	46,587 (10.53)	42,475 (9.43)	42,902 (8.40)	177,236 (9.57)	
	Year 2: EPIC Wellness Registry	0 (0)	3 (0)	5,596 (1.24)	11,079 (2.17)	16,678 (0.90)	
**Clinical characteristics, mean (SD)^c^**							
	Number of Chronic Conditions	2.3 (2.7)	2.4 (2.8)	2.4 (2.8)	2.2 (2.7)	2.3 (2.7)	
	Number of Medication Active Ingredients	2.2 (6.1)	2.2 (6.1)	2.3 (6.3)	2.1 (5.9)	2.2 (6.1)	
**Resource Utilization Band, n (%)^c^**							
	No or only invalid diagnosis	2,2507 (5.02)	20,273 (4.58)	16,897 (3.75)	35,288 (6.91)	94,965 (5.13)	
	Healthy users	44,731 (9.97)	43,408 (9.81)	40,996 (9.1)	53,142 (10.4)	182,277 (9.84)	
	Low resource use	59,400 (13.24)	55,221 (12.48)	57,667 (12.8)	69,566 (13.62)	241,854 (13.06)	
	Moderate resource use	232,748 (51.88)	231,680 (52.37)	239,473 (53.17)	257,424 (50.4)	961,325 (51.9)	
	High resource use	59,979 (13.37)	60,841 (13.75)	62,740 (13.93)	63,338 (12.4)	246,898 (13.33)	
	Very high resource use	29,301 (6.53)	30,943 (6.99)	32,653 (7.25)	32,012 (6.27)	124,909 (6.74)	

^a^Neighborhood characteristics for the person’s residence of the longest duration are reported as a percentile of national rank [30].

^b^ICD-10: *International Classification of Diseases, Tenth Revision*.

^c^These clinical measures are derived from the Johns Hopkins Adjusted Clinical Group (ACG) System version 12.0. The Resource Utilization Band represents expected future use based on current morbidities [29].

### GEE Modeling

Details of the GEE models are presented in Table 3. The GEE model had an area under the curve (AUC) of 0.702 (95% CI 0.699-0.705) in predicting prospective social needs in the overall study population. The strongest predictors of future social needs in the whole population in descending order were social needs documented in the EHR during the previous year period (odds ratio [OR] 3.285, 95% CI 3.237-3.335), ≥1 emergency department visit in the previous periods (OR 1.659, 95% CI 1.634-1.684), and a very high Resource Utilization Band measure indicative of a significant morbidity burden (OR 1.371, 95% CI 1.317-1.427).

**Table 3:** Generalized estimating equation model predicting prospective social needs for patients at Johns Hopkins Health System using electronic health record data between 2016 and 2021: overall model and models for selected subpopulations.

Variable			Overall Population	Population aged ≥65 years	Racial groups		Neighborhood characteristics	
					White	Black	Most disadvantaged	Least disadvantaged
Area under the curve (95% CI)			0.702 (0.699-0.705)	0.701 (0.696-0.706)	0.689 (0.685-0.693)	0.711 (0.706-0.716)	0.711 (0.708-0.714)	0.667 (0.663-0.677)
Age (years), OR^a^ (95% CI)			0.994 (0.993-0.994)	1.002 (1.001-1.004)	0.994 (0.994-0.995)	0.993 (0.992-0.994)	0.991 (0.991-0.992)	1.002 (1.001-1.002)
Gender (male; reference: female), OR (95% CI)			0.993 (0.981-1.004)	0.922 (0.903-0.941)	0.964 (0.95-0.979)	1.068 (1.046-1.092)	1.004 (0.991-1.017)	0.952 (0.927-0.977)
Race (Black; reference: White), OR (95% CI)			1.125 (1.11-1.141)	1.149 (1.118-1.182)	—^b^	—	1.127 (1.111-1.144)	1.137 (1.087-1.190)
Preferred language (English; reference: missing, others, or sign language), OR (95% CI)			1.061 (1.026-1.097)	1.293 (1.221-1.371)	1.034 (0.991-1.08)	1.112 (1.043-1.187)	1.030 (0.993-1.069)	1.217 (1.125-1.317)
Interpreter needed (yes; reference: no or missing), OR (95% CI)			1.179 (1.122-1.238)	1.266 (1.156-1.386)	1.006 (0.908-1.114)	0.816 (0.679-0.981)	1.148 (1.089-1.211)	1.085 (0.944-1.247)
Area deprivation index national rank (percentile), OR (95% CI)^c^			1.005 (1.005-1.005)	1.001 (1.001-1.002)	1.003 (1.003-1.004)	1.007 (1.006-1.007)	1.006 (1.006-1.006)	0.983 (0.979-0.988)
**Health care use, OR (95% CI)**								
	Any in-patient admission	1.017 (0.993-1.042)		1.077 (1.03-1.126)	1.064 (1.03-1.1)	0.987 (0.947-1.029)	0.986 (0.960-1.013)	1.131 (1.071-1.194)
	Any emergency department visits	1.659 (1.634-1.684)		1.539 (1.495-1.584)	1.691 (1.656-1.728)	1.627 (1.587-1.668)	1.636 (1.608-1.664)	1.555 (1.503-1.608)
Previous social needs, OR (95% CI)			3.285 (3.237-3.335)	3.043 (2.96-3.128)	3.459 (3.391-3.529)	2.9 (2.824-2.977)	3.390 (3.334-3.447)	2.775 (2.677-2.877)
**Clinical characteristics, OR (95% CI)^d^**								
	Number of chronic conditions	1.066 (1.064-1.069)		1.08 (1.07-1.08)	1.070 (1.067-1.074)	1.063 (1.058-1.068)	1.070 (1.067-1.073)	1.066 (1.058-1.073)
	Number of medication active ingredients	0.997 (0.996-0.998)		0.99 (0.99-1.00)	0.997 (0.995-0.998)	0.997 (0.995-0.999)	0.996 (0.995-0.997)	1.001 (0.999-1.004)
**Resource Utilization Band** **(reference:** **no** **or** **only** **invalid** **diagnosis), OR (95% CI)^d^**								
	Healthy users	0.838 (0.808-0.869)		0.792 (0.734-0.854)	0.836 (0.798-0.876)	0.785 (0.729-0.844)	0.837 (0.802-0.872)	0.787 (0.729-0.850)
	Low resource use	0.855 (0.826-0.885)		0.837 (0.778-0.9)	0.856 (0.819-0.895)	0.846 (0.792-0.904)	0.849 (0.816-0.882)	0.847 (0.787-0.911)
	Moderate resource use	0.964 (0.935-0.994)		0.935 (0.876-0.997)	0.942 (0.906-0.981)	1.008 (0.952-1.069)	0.978 (0.945-1.012)	0.908 (0.848-0.972)
	High resource use	1.259 (1.217-1.302)		1.226 (1.144-1.314)	1.238 (1.184-1.294)	1.328 (1.246-1.415)	1.285 (1.238-1.335)	1.113 (1.030-1.204)
	Very high resource use	1.371 (1.317-1.427)		1.218 (1.128-1.315)	1.291 (1.225-1.361)	1.514 (1.408-1.629)	1.420 (1.359-1.485)	1.136 (1.034-1.249)

^a^OR: odds ratio.

^b^Not available.

^c^Neighborhood characteristics for the person’s residence of longest duration are reported as a percentile of national rank [30].

^d^These clinical measures are derived from the Johns Hopkins Adjusted Clinical Group (ACG) System version 12.0. The Resource Utilization Band represents expected future use based on current morbidities [29].

To help assess bias and applicability to various subpopulations, we identified comparable performance for models of select subgroups, with AUCs of 0.701 (95% CI 0.696-0.706), 0.711 (95% CI 0.706-0.716), and 0.711 (95% CI 0.708-0.714), respectively, for individuals aged 65 years or older, Black patients, and those living in the most disadvantaged neighborhoods. The strongest predictor of future social needs in the study subpopulations remained the previous social needs, with ORs of 3.043 (95% CI 2.96-3.128), 2.9 (95% CI 2.824-2.977), and 3.390 (95% CI 3.334-3.447) among individuals aged 65 years or older, Black patients, and those living in the most disadvantaged neighborhoods, respectively.

To ensure that the most common social needs (ie, safety and economic challenges) were not the main drivers of the model’s performance, we performed a sensitivity analysis and ran the model after excluding patients with any social needs in subdomains of safety and economic challenges. This resulted in a slightly better-performing model with an AUC of 0.768 (95% CI 0.763-0.773). In this instance, previous social needs were by far the most significant predictor (OR 11.857, 95% CI 11.521-12.202), followed by emergency department visits (OR 1.916, 95% CI 1.865-1.969), need for an interpreter (OR 1.528, 95% CI 1.397-1.672), and Black race (OR 1.307, 95% CI 1.273-1.342). Contrary to the analyses performed with all social needs included, patients with higher Resource Utilization Band had slightly less risk of increased social needs, and lower Resource Utilization Band had slightly greater protective value regarding social needs (Table S2 in Multimedia Appendix 1 provides details of the GEE model).

## Discussion

### Overview

Achieving a comprehensive assessment of a person’s health and addressing health disparities goes beyond just documenting clinical diseases and medical interventions. We must also capture, standardize, analyze, and report reliable information on social needs and SDOH challenges within operational clinical decision support systems that are built into EHRs. Moreover, the rapid change toward “value-based” health care models [32] in the United States has required the incorporation of social needs and SDOH contexts and frameworks to ensure that the health care systems and health plans equitably address the needs of minority and disadvantaged communities [33,34]. For these value-based models to perform well, it is critical that clinical and social interventions are aligned and that no financial disincentives are imposed on providers who disproportionately serve minority and disadvantaged patients [33,34].

To achieve these goals, applied research is needed to identify optimal solutions for the effective collection and application of social needs and SDOH information within EHRs, link provider-based data to community-level data describing the characteristics of patients’ neighborhoods, and anchor such information to the providers’ digital workflow. This approach will provide the vehicle for harnessing social needs and SDOH data to target interventions at the point of care (eg, referrals of an individual); the health delivery system level (eg, hiring a social worker in the clinic); or the community (eg, building or strengthening community-based initiatives) [35]. To avoid burdensome and inefficient social needs assessment and data collection, it is essential to develop automated screening tools using EHR or community-based data to help identify the subset of patients who would most benefit from social needs assessment and data collection.

Several EHR-based screening tools for social needs assessment have been piloted in recent years, and results have shown these tools to be effective in determining social needs and SDOH challenges [36,37]. However, the feasibility of such tools remains unclear, with health care systems needing to dedicate considerable time and budget to train and educate staff and manage workloads [21]. Our proposed social risk score aimed to reduce the burden of this process and increase its accuracy by identifying patients at high risk of having any social needs for more efficient screening.

### Comparison With Previous Evidence

Our proposed model was based on a large and diverse data set of patients in the JHHS-EHR. The AUC of our model in predicting prospective social needs was 0.702 (95% CI 0.699-0.705) in the overall study population. This AUC may be the result of many instances of false negatives related to the documentation of social needs in structured EHR data. At the time of completing this study, social needs screening and referral were not common practices at our institutions. Thus, we expect many patients with social needs did not get a proper screening and documentation of such needs. The new mandate established by the Centers for Medicare and Medicaid Services requires hospitals to report to the Inpatient Quality Reporting Program 2 brand new measures of social needs (ie, the number of patients screened for social needs and the number of patients identified with selected social needs) [38]. We expect the health care system to establish more systematic and uniform processes for screening and documentation of social needs. This effort will increase the volume of data on social needs in the EHR, which will result in better performance of our models in the future.

Our findings were comparable with those in the study by Holcomb et al [14], where they predicted health-related social needs using EHR and community-level data and machine learning modeling for Medicare and Medicaid beneficiaries participating in the Accountable Health Communities project. Their models performed relatively well, with AUCs ranging from 0.59 to 0.68 for patients with different domains of social needs. Another notable mention was the study by Byrne et al [15], where they used EHR data, including responses to the Veterans Health Administration’s Homelessness Screening Clinical Reminder Survey, to develop and test predictive models of housing instability and homelessness. All their models performed well, with the random forest models performing better than the logistic regression models for both the housing instability (85.4 vs 78.3) and homeless (91.6 vs 87.1) outcomes. Lastly, Kasthurirathne et al [13] built random forest decision models to predict the need for social work referrals using clinical and population-level data on SDOH challenges. The performance of the model ranged from an AUC of 0.713 for the model using both clinical and SDOH data to 0.731 for the model using clinical data.

Moreover, our results demonstrated that the most significant predictive factor for having prospective social needs was the documentation of previous social needs. This study also found associations between prospective social needs with previous ED visits and morbidity-related high resource use presented in Resource Utilization Band. Lastly, our model showed a minimally increased risk of social needs in association with characteristics of the neighborhood of residence, presented as the ADI measure. This finding was similar to the results of the study by Nguyen et al [39], where they identified a small statistically significant association between the ADI and total score on social needs from the Health Leads Social Needs Survey among pediatric patients‘ families receiving primary care at a large academic institution. A review by Chen et al [21] also indicated the low success of the integration of population-level data for predictive modeling and risk stratification purposes, including the prediction of social-related service referrals [13], in contrast to the performance of models using individual-level data in referrals to a social worker [40]. Overall, these findings indicated that individual characteristics played a more crucial role in predicting future social needs than neighborhood characteristics.

### Clinical Implications

The implementation of an EHR-based social risk score such as the one we developed would have many implications for clinicians and practice organizations. At the point of care, our social risk score could be integrated directly with EHR-derived data warehouses; thus, the proposed risk score could be leveraged to allow clinicians to tailor more personalized care and modulate care coordination efforts. This personalized social risk score could also help support tools tailored to the needs of patients, which would empower the navigation of available social services [35]. At the health delivery system level, the linkages of geo-derived databases could improve the assessment of social needs and SDOH challenges for health systems and provide opportunities for longer-term plans to address those factors in current and future care management programs. Additionally, the merged clinical and nonclinical databases could enable providers to follow patients with social needs and SDOH challenges over time through their interaction with the health system. At the community level, the social risk score and the social needs or SDOH data could enhance care coordination efforts by integrating community-level data into clinical decision support tools and coordinated interventions [41].

### Limitations

Several limitations existed for this study. First, EPIC Wellness Registry data were essentially nonexistent between the years 2016 and 2018, which, in addition to the possibility of ICD-10 codes being underused by providers, might lead to an underrepresentation of social needs in this study’s population. Second, Latino and Hispanic patients were underrepresented in this study’s population, which may have impacted the generalizability of the proposed model. Furthermore, data on racial and ethnic minority groups such as American Indian and Alaskan Native, Native Hawaiian, Other Pacific Islander, and multiracial individuals were limited in our data set. Another important factor that might have affected our results was the potentially more frequent screening of social needs in female individuals, ethnic and racial minority populations, those with higher disease burdens, and superusers of health care services [42], leading to biased results for these individuals. Misclassification and inconsistency in documenting social needs in the EHRs could influence our results.

Moreover, the use of EHR data as the sole source of information limited our data to services provided to patients across the JHHS facilities and did not include other services outside JHHS. Also, we used the patient’s home address to link the EHR data to the American Community Survey community-level data. Thus, we did not include patients with a missed or invalid home address. This may have resulted in missing some patients with social needs, such as residential instability. Finally, our final model was applied to the 2020-2021 cohort to evaluate its accuracy, which included patient encounters during the first peak of the COVID-19 pandemic. While some subpopulations of patients experienced more social needs during the pandemic, the challenges that the health care systems faced during this period impacted the screening and documentation of social needs and may have resulted in missed data on social needs for this cohort.

### Conclusion

Screening for social needs and SDOH challenges using available secondary EHR data represents an important step toward addressing health disparities more efficiently and improving patient care and population health. Our proposed model integrates community-level data with patient-level data to arrive at a social risk score that can be used to systematically identify patients at increased risk of having future social needs and is thus appropriate for in-depth assessment of their social needs and potential referral to community-based organizations to address these needs. Our model identified previous social needs and high morbidity levels as the strongest predictors of future social needs. Missing data in the EHR reflecting past clinician documentation of social needs may have impacted the performance of our proposed model, and the predictive accuracy of models like ours will likely increase as the capture of such information becomes more commonplace. Future studies should focus on developing EHR-integrated clinical decision support tools to make this information available in the providers’ digital workflow and at the point of care.

## Appendix

Multimedia Appendix 1 Social needs domains across study cohorts and sensitivity analysis.

## Abbreviations

ACG: Adjusted Clinical Group
ADI: area deprivation index
AUC: area under the curve
EHR: electronic health record
GEE: generalized estimating equation
ICD-10: International Classification of Diseases, Tenth Revision
JHHS: Johns Hopkins Health System
OR: odds ratio
SDOH: social determinants of health

## Data Availability

The data sets generated and analyzed during this study are not publicly available due to concerns regarding the privacy of individuals whose electronic health records information were used.
