# Supplementary material for: Development of a Social Risk Score in the Electronic Health Record to Identify Social Needs Among Underserved Populations: Retrospective Study
Source: JMIR Form Res. 2024 Mar 12;8:e54732. doi: 10.2196/54732 (PMC10966439; doi:10.2196/54732)
Supplement: Multimedia Appendix 1 [file formative_v8i1e54732_app1.docx]

| **Table S1. Distribution of Social Needs by Different Domains and Subdomains Among the Study Population Using Electronic Health Record Data at Johns Hopkins Health System Between 2016-2021***** | | | | | |
| --- | --- | --- | --- | --- | --- |
| **Social Needs** | **Study Cohort** | | | | **Overall** |
|  | **2016-2017** | **2017-2018** | **2018-2019** | **2019-2020** |  |
| **Social** | **24,533 (5.47)** | **27,174 (6.14)** | **29,393 (6.53)** | **30,950 (6.06)** | **112,050 (6.05)** |
| Safety | 19,211 (4.28%) | 21,400 (4.84%) | 22,886 (5.08%) | 23,655 (4.63%) | 87,152 (4.71%) |
| Social Connection | 989 (0.22%) | 1,448 (0.33%) | 1,749 (0.39%) | 2,487 (0.49%) | 6,673 (0.36%) |
| Stress | 5,387 (1.20%) | 5,702 (1.29%) | 6,279 (1.39%) | 6,638 (1.30%) | 24,006 (1.30%) |
| Race/ethnicity | 0 | 0 | 1 (0%) | 1 (0%) | 2 (0%) |
| Migration | 6 (0%) | 6 (0%) | 17 (0%) | 40 (0.01%) | 69 (0%) |
| Incarceration | 92 (0.02%) | 102 (0.02%) | 161 (0.04%) | 166 (0.03%) | 521 (0.03%) |
| Military | 0 | 0 | 0 | 0 | 0 |
| **Education** | | | | | |
| Education | 162 (0.04%) | 215 (0.05%) | 269 (0.06%) | 217 (0.04%) | 863 (0.05%) |
| **Economic** | **6,435 (1.43%)** | **18,690 (4.23%)** | **17,373 (3.86%)** | **15,744 (3.08%)** | **58,242 (3.14%)** |
| Financial Resource Strain | 26 (0.01%) | 42 (0.01%) | 90 (0.02%) | 136 (0.03%) | 294 (0.02%) |
| Employment | 6,010 (1.34%) | 18,218 (4.12%) | 16,940 (3.76%) | 15,335 (3.00%) | 56,503 (3.05%) |
| Nutrition and Food Insecurity | 417 (0.09%) | 475 (0.11%) | 392 (0.09%) | 340 (0.07%) | 1,624 (0.09%) |
| **Healthcare System** | | | | | |
| Access to Health Services | 7,350 (1.64%) | 8,049 (1.82%) | 8,997 (2.00%) | 9,193 (1.80%) | 33,589 (1.81%) |
| **Physical Environment** | | | | |  |
| Residential Instability | 2,632 (0.59%) | 3,175 (0.72%) | 3,531 (0.78%) | 3,730 (0.73%) | 13,068 (0.71%) |
| *** Numbers (%) reported based on the documented ICD-10 codes in the first year of each cohort. | | | | | |

| **Table S2. Generalized Estimating Equation Model Predicting Prospective Social Needs for Patients at Johns Hopkins Health System Using Electronic Health Record Data Between 2016-2021: Sensitivity Analysis After Removing two Most Common Social Needs Subdomains.**^*^ | |
| --- | --- |
| **Variable** | **Odds Ratio (95% Confidence Interval)** |
| **Area Under the Curve** | |
|  | 0.768 (0.763-0.773) |
| **Age** – Years | |
|  | 0.989 (0.989-0.990) |
| **Gender** – Male (ref: female) | |
|  | 1.032 (1.009-1.055) |
| **Race** – Black (ref: White) | |
|  | 1.307 (1.273-1.342) |
| **Preferred Language** – English (ref: missing, others or sign language) | |
|  | 1.149 (1.074-1.230) |
| **Interpreter Needed** – Yes (ref: no or missing) | |
|  | 1.528 (1.397-1.672) |
| **Area Deprivation Index National Rank** – Percentile | |
|  | 1.008 (1.008-1.008) |
| **Healthcare Utilization** | |
| *Any In-Patient Admission* | 1.113 (1.067-1.161) |
| *Any ED Visits* | 1.916 (1.865-1.969) |
| **Previous Social Needs** | |
|  | 11.857 (11.521-12.202) |
| **Clinical Characteristics** – mean (standard deviation)^†^ | |
| *No. of Chronic Conditions* | 1.065 (1.060-1.070) |
| *No. of Medication Active Ingredients* | 0.990 (0.988-0.992) |
| **Resource Utilization Bands** (ref: no or only invalid diagnosis)^†^ | |
| *Healthy users* | 0.596 (0.556-0.639) |
| *Low resource utilization* | 0.628 (0.589-0.668) |
| *Moderate resource utilization* | 0.713 (0.675-0.753) |
| *High resource utilization* | 0.956 (0.901-1.016) |
| *Very high resource utilization* | 1.097 (1.022-1.178) |
| * Odds ratios (and 95% confidence intervals) are presented in the table. The model includes all covariates but has removed the two most common social needs subdomains (i.e., safety and economic challenges).  † These clinical measures are derived from the Johns Hopkins Adjusted Clinical Group (ACG) System Version 12.0. Resource Utilization Band represents expected future utilization based on current morbidities.[29]. | |
